# A NEW LOOK AT NEUROTICISM: FINDINGS FROM THE IOWA UNMARRIED SURVIVORS STUDY

**DOI:** 10.1093/geroni/igae098.2500

**Published:** 2024-12-31

**Authors:** Robert Hensley, Peter Martin, Alex Bishop

**Affiliations:** Southeast Community College, Lincoln, Nebraska, United States; Iowa State University, Ames, Iowa, United States; Oklahoma State University, Stillwater, Oklahoma, United States

## Abstract

The purpose of this study was to analyze the association between personality traits and other variables relative to Neuroticism reported by N = 227 (M = 78.2, SD = 8.1) participants from the Iowa Unmarried Survivors Study. Using the development adaptation model as a conceptual framework (and Neuroticism as the dependent variable), a series of blocked multiple regression analyses were conducted. In the first block, age, gender, ethnicity, total years of education, and current marital status were included. The second block contained selected childhood variables, whereas the third block contained the personality variables, and the fourth block included the Venting subscale of the Brief COPE, social support, depression, stress, and loneliness. Recalled childhood abuse emerged as a significant predictor of Neuroticism, β =.118, p <.05. If one was abused in childhood, one would show higher levels of Neuroticism. In addition, both venting and loneliness were significant predictors of Neuroticism, β =.120, p <.05, β =.191, p <.05 respectively. Moreover, stress and depression served as significant predictors of Neuroticism, β =.208, p <.01, β =.432, p <.01 respectively. This model explained 64% of the variance in Neuroticism scores. Although a causal relationship cannot be established via this model, the mulitdirectional nature of Neuroticism is noteworhy. Results from this study have implications relative to how geriatric social workers and aging service providers develop interventions and programming to improve mental health and quality of life for persons who remain unmarried throughout later life.

